# Cortical microvascular blood flow velocity mapping by combining dynamic light scattering optical coherence tomography and two-photon microscopy

**DOI:** 10.1117/1.JBO.28.7.076003

**Published:** 2023-07-21

**Authors:** Qi Pian, Mohammed Alfadhel, Jianbo Tang, Grace V. Lee, Baoqiang Li, Buyin Fu, Yagmur Ayata, Mohammad Abbas Yaseen, David A. Boas, Timothy W. Secomb, Sava Sakadzic

**Affiliations:** a Massachusetts General Hospital, Harvard Medical School, Athinoula A. Martinos Center for Biomedical Imaging, Department of Radiology, Charlestown, Massachusetts, United States; b Northeastern University, Department of Bioengineering, Boston, Massachusetts, United States; c Southern University of Science and Technology, Department of Biomedical Engineering, Shenzhen, China; d University of Arizona, Program in Applied Mathematics, Tucson, Arizona, United States; e Chinese Academy of Sciences, Shenzhen Institute of Advanced Technology, Brain Cognition and Brain Disease Institute; Shenzhen Fundamental Research Institutions, Shenzhen–Hong Kong Institute of Brain Science, Shenzhen, Guangdong, China; f Boston University, Department of Biomedical Engineering, Boston, Massachusetts, United States; g University of Arizona, Department of Mathematics, Tucson, Arizona, United States; h University of Arizona, Department of Physiology, Tucson, Arizona, United States

**Keywords:** cerebral blood flow, dynamic light scattering, optical coherence tomography, two-photon microscopy, image coregistration, microvascular angiography

## Abstract

**Significance:**

The accurate large-scale mapping of cerebral microvascular blood flow velocity is crucial for a better understanding of cerebral blood flow (CBF) regulation. Although optical imaging techniques enable both high-resolution microvascular angiography and fast absolute CBF velocity measurements in the mouse cortex, they usually require different imaging techniques with independent system configurations to maximize their performances. Consequently, it is still a challenge to accurately combine functional and morphological measurements to co-register CBF speed distribution from hundreds of microvessels with high-resolution microvascular angiograms.

**Aim:**

We propose a data acquisition and processing framework to co-register a large set of microvascular blood flow velocity measurements from dynamic light scattering optical coherence tomography (DLS-OCT) with the corresponding microvascular angiogram obtained using two-photon microscopy (2PM).

**Approach:**

We used DLS-OCT to first rapidly acquire a large set of microvascular velocities through a sealed cranial window in mice and then to acquire high-resolution microvascular angiograms using 2PM. The acquired data were processed in three steps: (i) 2PM angiogram coregistration with the DLS-OCT angiogram, (ii) 2PM angiogram segmentation and graphing, and (iii) mapping of the CBF velocities to the graph representation of the 2PM angiogram.

**Results:**

We implemented the developed framework on the three datasets acquired from the mice cortices to facilitate the coregistration of the large sets of DLS-OCT flow velocity measurements with 2PM angiograms. We retrieved the distributions of red blood cell velocities in arterioles, venules, and capillaries as a function of the branching order from precapillary arterioles and postcapillary venules from more than 1000 microvascular segments.

**Conclusions:**

The proposed framework may serve as a useful tool for quantitative analysis of large microvascular datasets obtained by OCT and 2PM in studies involving normal brain functioning, progression of various diseases, and numerical modeling of the oxygen advection and diffusion in the realistic microvascular networks.

## 1 Introduction

Cerebral blood flow (CBF) performs an essential role in satisfying the metabolic needs of the brain by transporting glucose and oxygen to the brain tissue and clearing metabolic waste, such as ${\mathrm{CO}}_{2}$, from it via a dense microvascular network. The crucial mechanism that accounts for continuously adjusting CBF to the local metabolic needs is defined as neurovascular coupling (NVC),^1^^,^^2^ and its status correlates with brain cognitive function.^3^^–^^5^ The NVC also plays a vital role in interpreting brain functioning using imaging techniques, such as functional magnetic resonance imaging and positron emission tomography.^6^^,^^7^ The NVC relies on the coordination of multiple signaling pathways involving various cell types in the brain, such as neurons, astrocytes, pericytes, as well as vascular smooth muscle cells and endothelial cells.^8^^,^^9^ On the other hand, NVC can be impaired under various pathological conditions, such as Alzheimer’s disease,^10^^–^^13^ hypertension,^14^ atherosclerosis,^15^ and seizures.^16^ Therefore, the accurate mapping of CBF velocities from a dense microvascular network could be a key step toward a better understanding of NVC in both normal and pathological brain conditions. In addition, it could also contribute to the development of more accurate numerical models of oxygen delivery and consumption in realistic microvascular networks.^17^^,^^18^

A suitable method to achieve this task should be capable of (i) obtaining accurate cerebrovascular angiograms with microscopic resolution that will include a majority of or all arterioles, venules, and capillaries over a large field of view (FOV) and (ii) acquiring volumetric blood flow velocity data from the majority of the microvascular segments over the same FOV. Several imaging modalities, such as multiphoton microscopy (MPM)^19^^–^^21^ [e.g., two-photon microscopy (2PM) and three-photon microscopy (3PM)], optical coherence tomography (OCT),^22^^–^^25^ photoacoustic microscopy (PAM),^26^^–^^29^ and 3D ultrasound localization microscopy (ULM)^30^^–^^33^ are capable of acquiring both microvascular angiograms and blood flow in most cortical microvascular segments. Among these imaging methods, MPM is unique in its ability to provide more accurate morphological parameterization of the microvascular networks *in vivo*. It is the preferred method to acquire accurate capillary morphology *in vivo*, across the mouse cortical layers (∼1  mm-thick) and over a large FOV (up to $1\times 1\;{\mathrm{mm}}^{2}$). On the other hand, MPM, OCT, PAM, and ULM are all capable of acquiring large microvascular velocity maps in a short time, which is very important for *in vivo* imaging studies. MPM typically employs a line scan method to measure the blood flow velocity that is perpendicular to the optical axis direction for arteries, veins, and capillaries. For each vessel segment, the line scan is performed repeatedly at a 1 to 2 kHz rate along its longitudinal direction until it is sufficient to determine the flow velocity, and ∼40  ms is a reasonable target for measurement temporal resolution,^19^ which renders it only suitable for retrieving cerebral flow velocity values from a limited number of vessel segments. Faster volumetric scanning MPM has been enabled by raster-scanning a Bessel beam^34^ and blood flow velocities can be rapidly acquired with this method over a limited FOV.^35^ Recently, an ultrafast free-space angular chirp enhanced delay scanning protocol has increased the temporal resolution for two-photon cortical blood flow imaging by orders of magnitude to ∼1  kHz 2D frame rate.^36^ PAM is a label-free method for wide-FOV (e.g., several ${\mathrm{mm}}^{2}$) and relatively deep (e.g., ∼1  mm imaging depth) mapping of total hemoglobin concentration ($C_{\mathrm{Hb}}$), oxygen saturation of hemoglobin (${\mathrm{sO}}_{2}$), and blood flow speed by making use of the absorption contrast, weak acoustic scattering in soft tissue, and high optical resolution (lateral: 2 to 3  μm and axial: ∼15  μm).^37^ Fast 3D volumetric imaging at ∼1  Hz is achievable with PAM over a 4 to $6\;{\mathrm{mm}}^{2}$ FOV.^26^^,^^28^ ULM achieves super-resolution microvascular imaging by tracking microbubbles (diameter: 1 to 3  μm) administered into the bloodstream of the subjects and utilizing the super-resolution concept from optical microscopy. ULM is able to map the microvasculature from the entire mouse brain with data acquisition time of several tens of seconds, spatial resolution of ∼20  μm, and sensitivity to flow velocities between 2 and 100  mm/s.^33^ However, ULM requires the localization of individual microbubbles in the bloodstream. For imaging the small vessels, this is a time-consuming procedure and it may take tens of minutes to resolve the capillaries.^32^ OCT has been widely used for mapping absolute axial blood flow based on the Doppler effect that results from the motion of RBCs. The common Doppler OCT method usually acquires dense A-scans within a B-scan to resolve the phase change, and typical imaging time for such a B-scan is ∼40  ms.^22^^,^^23^ Capillary blood flow velocities can also be obtained by analyzing the dynamic scattering component of the OCT signals using the power spectrum bandwidth of the autocorrelation function^38^ or laser speckle decorrelation time.^39^ Recently, dynamic light scattering optical coherence tomography (DLS-OCT)^40^^–^^42^ was proposed to measure absolute blood flow velocity and the RBC diffusion coefficient. DLS-OCT can provide volumetric imaging over a FOV of $600\times 600\;\mu m^{2}$ and ∼1  mm imaging depth, with ∼3.5  μm isotropic spatial resolution and with ∼6.5-min-long data acquisition time for a single volume. This is achieved by utilizing the DLS approach for estimating RBC velocities and diffusion coefficients and has proved to be less susceptible to noise and more sensitive to RBC motion than Doppler OCT,^43^ leading to reliable blood flow velocity measurements in the capillaries. OCT is also capable of obtaining the microvascular angiograms^44^ but, similar to PAM and ULM, with inferior quality to the MPM angiograms, which renders it less optimal for accurate image segmentation and quantitative analysis of microvascular morphology. While multiple imaging modalities may be used to measure the microvascular CBF velocities, OCT has been among the most common tools applied for this task and it is optically highly compatible with MPM. Therefore, we selected a combination of DLS-OCT and 2PM to acquire both the CBF velocities from a large number of microvascular segments and high-resolution microvascular angiograms, respectively. It is worth noting that the framework developed based on the combination of DLS-OCT and 2PM can be extended to accommodate other possible multimodal imaging strategies, such as OCT and 3PM, which has gained traction over the past several years for acquiring angiograms with an increased imaging depth compared to 2PM. However, it is very difficult to integrate OCT and MPM together for simultaneous multimodal imaging without sacrificing their optimal performance. For example, objective lenses with high numerical aperture (NA) (NA>0.8) and properly filled back aperture by the laser beam are typically used in 2PM to achieve ∼1  μm lateral resolution and sufficient fluorescence emission signal collection efficiency, whereas using the same objective lens with a filled back aperture in an OCT system will create a de facto optical coherence microscope with extremely small depth of focus, which will prevent efficient acquisition of volumetric cerebral angiograms and blood flow velocity in mouse brain. In addition, MPM and OCT require significantly different scanning protocols, which are difficult to achieve if the excitation beams are scanned simultaneously by the same scanning mirrors. Although various attempts could be made to overcome these problems, such as drastically underfilling of the objective back aperture with the OCT beam to increase the depth of focus of OCT or using a Bessel beam to extend the OCT imaging range in the axial direction when using high-NA objective lenses, they will significantly complicate the imaging setup and make it much less practical. On the other hand, utilizing separate scanning mirrors for 2PM and OCT removes the constraints on scanning protocols, but it introduces different optical distortions to two imaging modalities and, therefore, requires advanced 3D coregistration.

In this work, we developed a computational framework to co-register large-scale microvascular blood flow velocity measurements from DLS-OCT with the high-resolution microvascular angiograms obtained using 2PM over the same FOV. We first performed a global 3D coregistration and a multilayer 2D coregistration to transform the 2PM angiogram onto the DLS-OCT angiogram. Then we segmented the original 2PM microvascular angiogram, obtained its mathematical graph representation, and transformed the vectorized angiogram onto the DLS-OCT image space based on the transformation matrices computed in the first step. Finally, the DLS-OCT flow velocity measurements were mapped onto the 2PM angiogram and mean microvascular segment velocities were extracted. The developed framework was implemented to process the datasets acquired from the three mice cortices. The distributions of mean CBF velocities in arterioles, venules, and capillaries as a function of vessel type and the branching order from precapillary arterioles and postcapillary venules have been retrieved. The framework proposed here will facilitate the numerical modeling of CBF and oxygen transport within cerebral microvascular networks and help us improve our understanding of the microvascular blood flow regulation in normal brain and in various brain conditions.

## 2 Methods

### 2.1 Animal Preparation

Three healthy, young-adult female C57BL/6 mice (∼3 months old; The Jackson Laboratory, Maine, United States) were used in this study. A sealed 3-mm-diameter cranial window was installed over the whisker barrel cortex area (on the left hemisphere; ∼2.0  mm posterior to bregma and ∼3.0  mm lateral from the midline). Tracheotomy was performed to enable controlled ventilation and maintain proper physiological status in subjects by providing a mixture of medical air (∼60  mL/min) and isoflurane (1.2% to 1.5%). A femoral artery catheter was installed to facilitate the administration of a contrast agent in 2PM imaging and to monitor physiological parameters, such as blood pressure, heart rate, and blood gases. Mice were kept under isoflurane anesthesia during the subsequent imaging sessions.

### 2.2 DLS-OCT Imaging

**Fig. 1:**
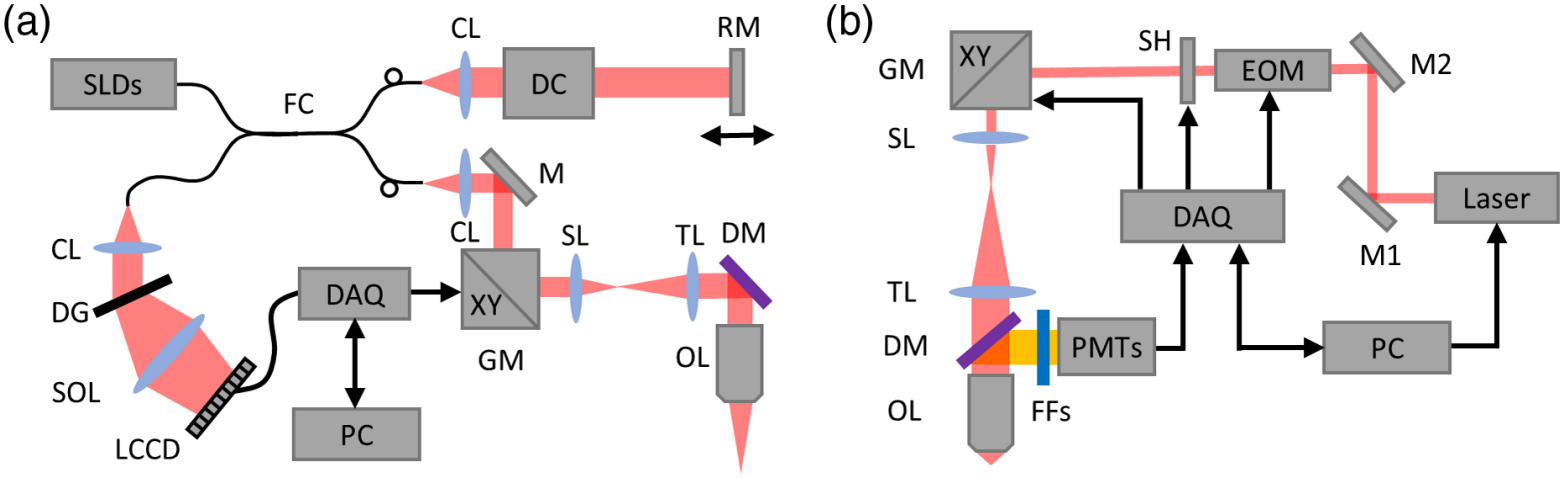
The diagrams of the OCT and 2PM imaging setups. (a) Diagram of the spectral domain OCT system. SLDs, superluminescent diodes; FC, fiber coupler; CLs, collimating lenses; DC, dispersion compensation; RM, reference mirror; M, mirror; GM, galvo mirrors; SL, scan lens; TL, tube lens; DM, dichroic mirror; OL, objective lens; DG, diffraction grating; SOL, spectrometer objective lens; LCCD, line-scan CCD; DAQ, data acquisition card; and PC, computer. (b) Diagram of the 2PM system. M1 and M2: mirrors; EOM, electro-optic modulator; SH, shutter; GM, galvanometer mirrors; SL, scan lens; TL, tube lens; DM, dichroic mirror; OL, objective lens; FFs, fluorescence filters; PMTs, photomultiplier tubes; DAQ, data acquisition card; and PC, computer.

DLS-OCT was performed using a spectral domain OCT setup^42^ (Thorlabs Inc., New Jersey, United States) [Fig. 1(a)]. The setup utilizes an extended broadband superluminescent diode (SLD) source (LS2000B, Thorlabs Inc., New Jersey, United States) that implements two matched-pair SLDs to offer >170  nm bandwidth at ∼1300  nm center wavelength. A 10× objective (MPlanApoNIR, NA=0.26, Mitutoyo, Japan) was used in the experiments. An InGaAs line scan camera with 1024 pixels was used to record the interference signal at 46,000 A-scans/s. The system can achieve ∼3.5  μm spatial resolution in all three dimensions within the brain tissue and ∼1  mm maximum imaging depth. OCT *en face* images of the pial surface were obtained for selecting a 600  μm×600  μm FOV (400  pixels×400  pixels). In this study, we only imaged down to ∼250  μm beneath the brain surface of each subject considering the ∼150  μm confocal parameter of the system since it provided a sufficient number of arterioles, venules, and capillaries to demonstrate the proposed framework. We first performed OCT angiography and repeated B-scan acquisition protocol was employed. The final 3D angiogram was obtained with 10 times averaging, which took 88 s for data acquisition. For the blood flow speed measurements using DLS-OCT, an M-mode data acquisition protocol with 100 A-scan repeats at each transverse location was employed and the data acquisition time for a 600  μm×600  μm×250  μm (voxel size: $1.5\times 1.5\times 2.9\;\mu m^{3}$) volume was ∼387  s. Since the measurement of a component of flow velocity that is parallel with the optical axis (Vz) has the largest SNR of all DLS-OCT velocity estimates, we relied on it to calculate the microvascular flow velocity based on the angle between vessel direction and the optical axis of the system.

### 2.3 2PM Angiography

**Fig. 2:**
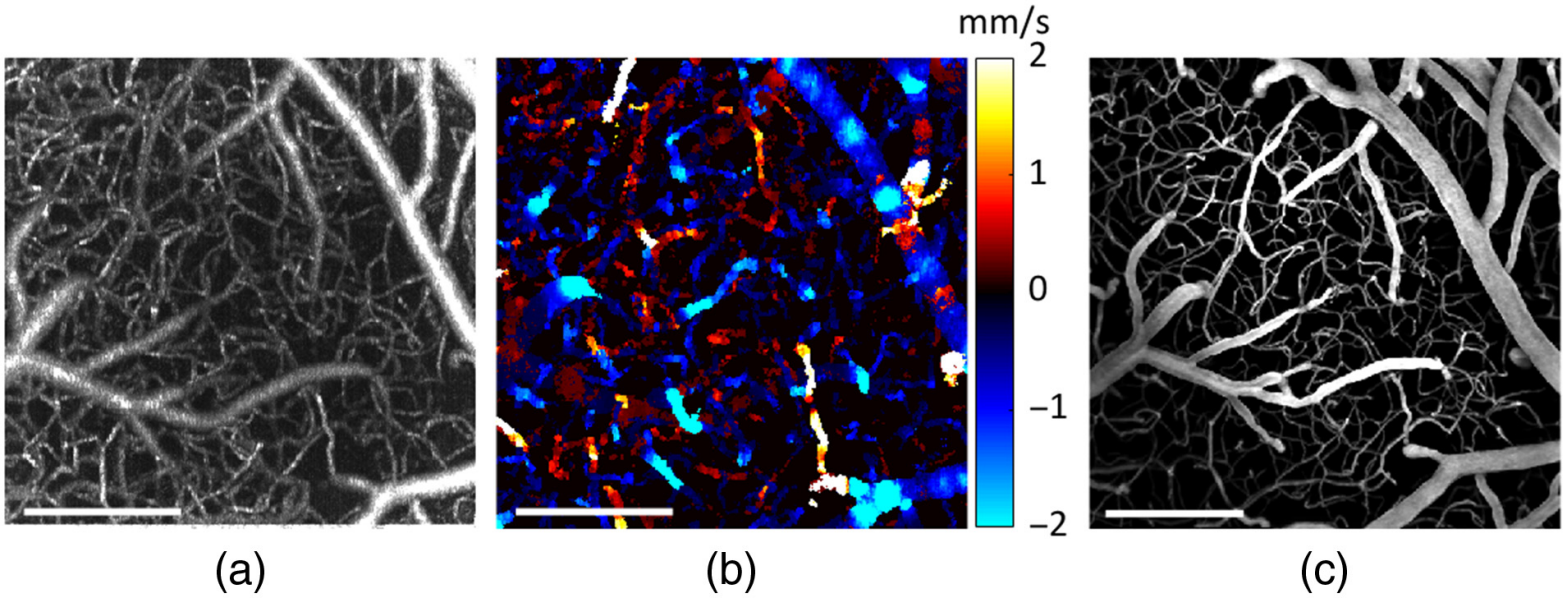
Top views of OCT and 2PM volumetric datasets acquired in one mouse. (a) Maximum intensity projection (MIP) of the OCT angiogram stack (FOV: 600  μm×600  μm). (b) Maximum projection of the DLS-OCT axial blood flow velocity Vz stack (FOV: 600  μm×600  μm). (c) MIP of the 2PM angiogram stack (FOV: 689  μm×689  μm). Scale bars: 200  μm.

The 2PM angiograms were acquired after DLS-OCT measurements using a custom-built 2PM setup^45^ [Fig. 1(b)]. A mode-locked laser (Insight DeepSee, ∼120  fs pulse width, 80 MHz pulse repetition rate, Spectra-Physics, California, Unites States) tunable between 680 and 1300 nm was used for the 2P excitation. The output power of the laser was controlled by an electro-optic modulator (EOM) (Model 350-160, ConOptics Inc., Connecticut, United States). Beam scanning in the XY plane was achieved by a set of galvanometer mirrors (6215H, Cambridge Technology Inc., Massachusetts, United States) with laser beam relayed by a scan lens (f=30  mm, AC254-030-B, Thorlabs Inc., New Jersey, United States) and a tube lens (f=180  mm, Olympus, Japan) to the back aperture of the objective. A water immersion objective lens (XLUMPLFLN20XW, NA = 1.00, Olympus, Japan) was used to focus the beam on the sample. The far-red fluorescent dye Alexa Fluor 680 conjugated to 70 kDa dextran was used to label blood plasma as the contrast agent for 2PM (400  μM, 0.1 mL). The emission signal of Alexa Fluor 680 was detected using a photomultiplier tube (PMT) (H10770PA-50, Hamamatsu, Japan), a dichroic mirror (FF875-Di01-25×36, Semrock, New York, United States), and two fluorescence filters (FF01-890/SP-50 and FF01-709/167-25, Semrock, New York, United States). In this study, 2PM survey images of the pial surface were first acquired and, with help of previously acquired OCT *en face* images, used to define an ROI (689  μm×689  μm) for 2PM that contained the ROI of DLS-OCT. The 2PM angiogram was subsequently acquired down to an ∼800  μm imaging depth (voxel size: $1.35\times 1.35\times 2.0\;\mu m^{3}$). The use of animals in this study was approved by the Institutional Animal Care and Use Committee at Massachusetts General Hospital. Typical examples of the volumetric datasets acquired from the two imaging sessions are visualized in Figs. 2(a)–2(c).

### 2.4 Angiogram Segmentation and Graphing

For quantitative modeling and analysis of cerebral microvascular networks, graph-based representations of the structure are needed.^46^^,^^47^ The 2PM angiograms were analyzed using Amira software (Thermo Fisher Scientific, Massachusetts, United States) combined with custom software written in C++, running on a graphical processor unit equipped computer, in the following steps. (i) *Adaptive contrast enhancement.* For each pixel in each image, the distribution of intensities in a surrounding 201×201 window were sampled to create a histogram and the intensities at the 50th and 99th percentiles ($I_{50}$ and $I_{99}$) were calculated. A linear mapping of intensities was performed, with $I_{50}$ mapped to zero and $I_{99}$ mapped to maximum intensity. This technique counteracts the effect of systematic intensity variations through the depth of the stack. (ii) *Vesselness filtering.* A novel 3D vesselness filter was developed. A set of 28 directions in space was defined, giving approximately uniform coverage of a hemisphere. For each direction, a test function of position was defined as $f=\exp[-1/2(x_{i}/\sigma_{x}{)}^{2}-1/2{\left(y_{i}/\sigma_{y}\right)}^{2}]$, where $x_{i}$ is the distance from the origin parallel to direction i, and $y_{i}$ is the distance perpendicular to direction i, and $\sigma_{x}=3$ and $\sigma_{y}=0.5\text{ pixels}$. For a given voxel, this function was convoluted with the image stack for each of the 28 directions, and the maximum result of these was used to construct the processed image at that voxel. This test function has an elongated prolate ellipsoidal distribution, and the procedure preferentially enhances narrow filamentous structures. Because of the small size of the test function, larger structures in the image are not significantly affected by this filter. Larger vessels generally show continuous intensity in the axial direction and do not need enhancement by vesselness filtering. (iii) *Fill-in filtering*. Some larger vessels show low intensity in their interior, with intensely labeled walls. To fill in such structures, the following method is used. From a given voxel, a set of six lines of 25 voxels extending in each (positive and negative) coordinate direction is defined, and the maximal intensity on each line is identified. If the mean of the six maxima exceeds the intensity at the given voxel, the intensity of the given voxel is increased, by an amount that decreases exponentially with the coefficient of variation of the six maxima. This method ensures that a voxel that is surrounded in multiple directions by voxels of higher intensity receives a maximal boost in intensity. (iv) *Segmentation.* Using Amira, the stack was thresholded to obtain a 3D solid representing the network. (v) *Skeletonization.* Using Amira, the solid was skeletonized, to yield a graph consisting of nodes and edges with defined diameters. (vi) *Refinement.* The skeleton was further processed to combine short, connected edges and to remove tight loops, which occur as artifacts of the skeletonization. The result is a representation of the network as a set of nodes and edges, with several edges forming a segment between vessel branching points. Segment diameters were estimated as the median of edge diameters in the segment. In this study, the graphs that represent the 2PM angiograms of three animal subjects were able to enclose in a connected network more than 99.9%, 99.3%, and 99.5% of the total vessel segments, respectively. A version of the custom software used is available online (https://github.com/secomb/StackEnhanceV1).

**Fig. 3:**
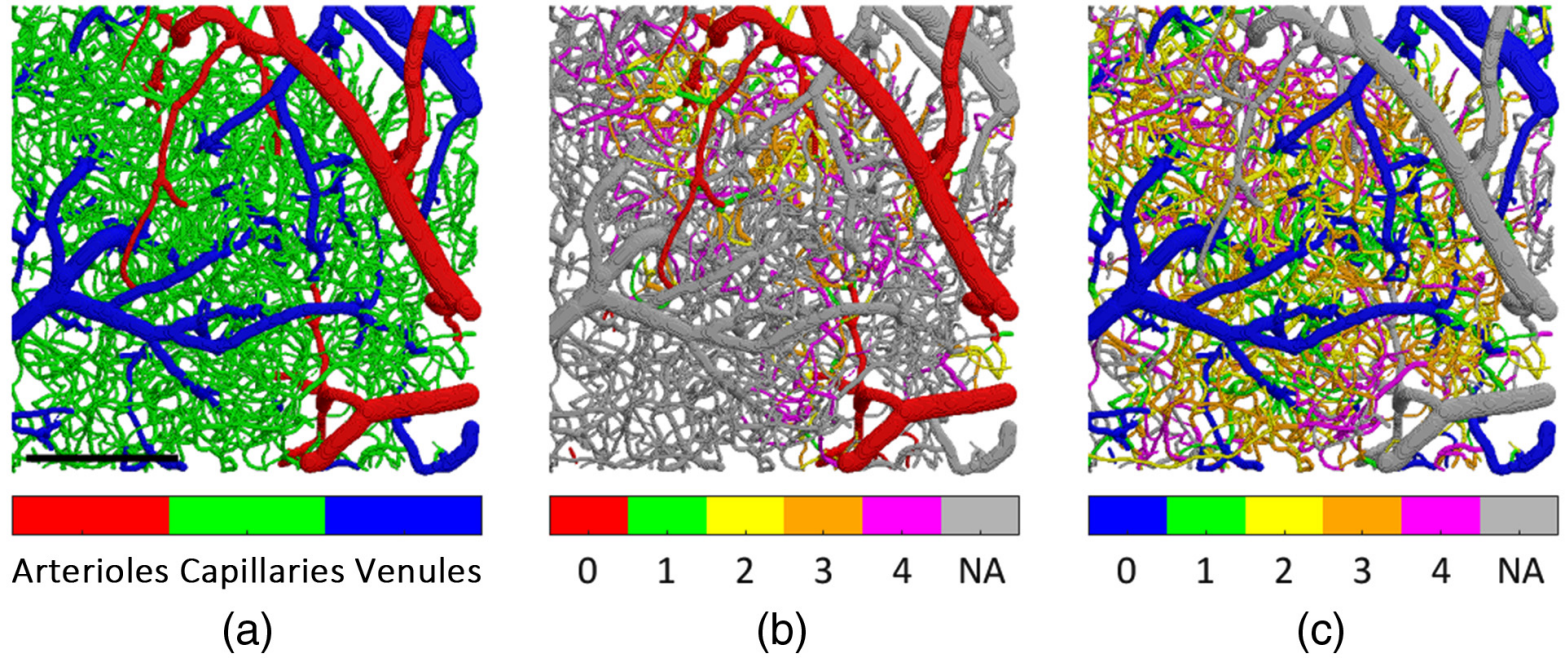
Angiogram segmentation and graphing results. (a) Overlay of vessel types on angiogram. Overlay of vessel branching order on angiogram from the (b) arteriolar side and (c) venular side.

The vessel type (e.g., arteriole, venule, or capillary) was associated with each segment by a combination of manual and automated vessel labeling.^48^^,^^49^ First, all pial arterioles and venules, as well as the initial segments of diving arterioles and ascending venules close to the pial surface were identified manually based on their morphology and flow direction in their branches that dive in and surface out of the cortex (e.g., penetrating arterioles and surfacing venules, respectively). Then the labeling of the remaining network was performed automatically starting from the labeled pial vascular segments and propagating the same label (e.g., arteriole or venule) down the vascular tree, excluding the microvascular segments that branch to a transverse plane. The remaining microvascular segments were labeled as capillaries. Finally, a color-coded mask of vessel types was generated based on the automatic labeling and overlaid on the angiogram stack for manual inspection and correction [Fig. 3(a)].

The capillary branching order starting from precapillary arterioles was computed by first assigning branching order zero to all pial and diving arterioles and subsequently assigning branching orders to the rest of the graph network starting from zero-order segments [Fig. 3(b)].^49^ The same procedure was then repeated for assigning the capillary branching order starting from the postcapillary venules [Fig. 3(c)].

### 2.5 Coregistration of the 2PM and OCT Angiograms

**Fig. 4:**
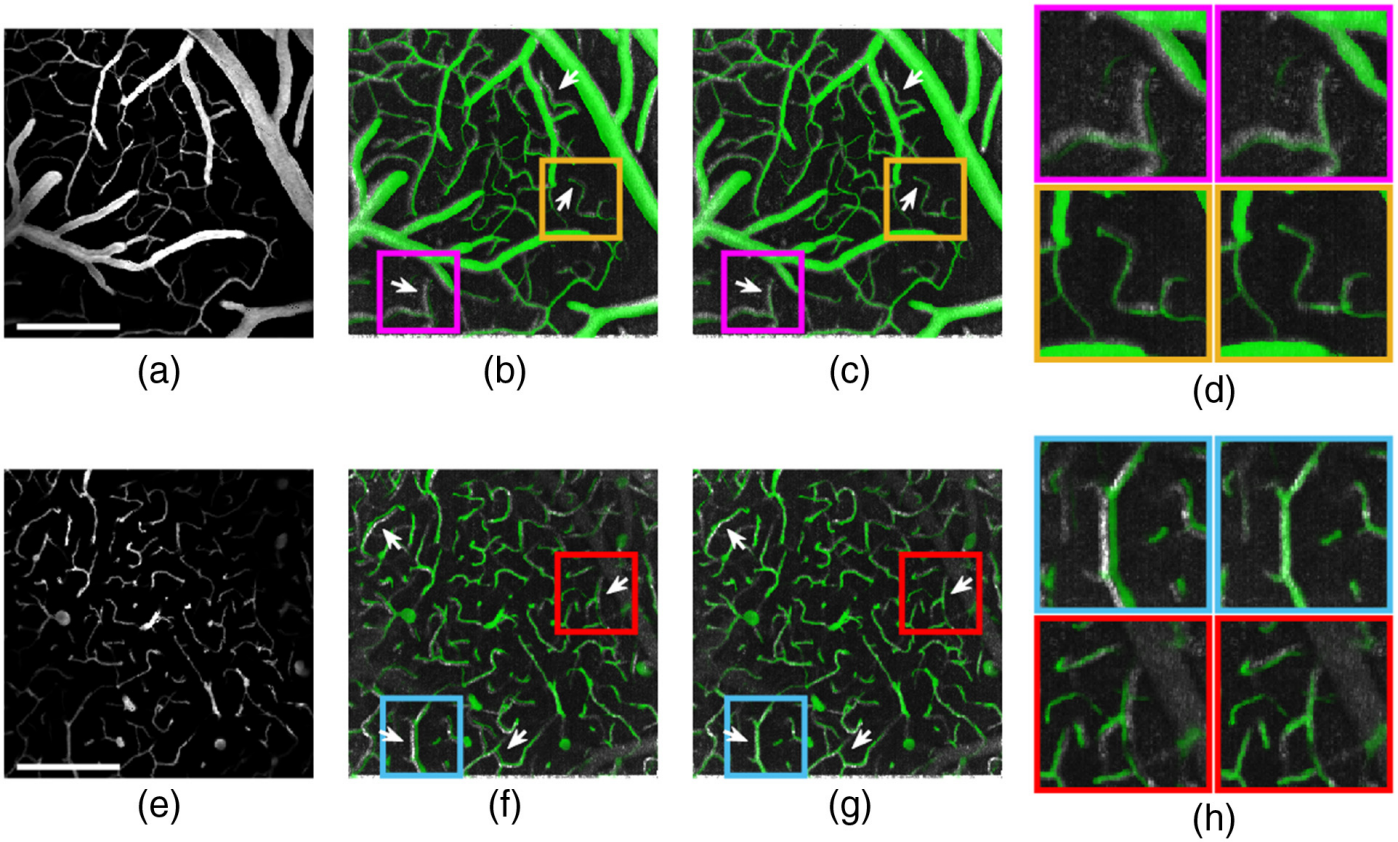
Angiogram coregistration results. Transformed 2PM pial stack (a) (depth range: 41 to 82  μm) in DLS-OCT space and corresponding coregistration results with DLS-OCT angiogram layers (gray) overlaid by the 2PM angiogram (green) using (b) global 3D coregistration only and (c) combined global 3D coregistration and 2D regional coregistration. (d) Zoomed views of two sub-areas in stack (a) that highlights the improvements with combined 3D and 2D coregistration. Transformed 2PM in-depth stack (e) (depth range: 157 to 197  μm) in DLS-OCT space and corresponding coregistration results with (f) global 3D coregistration only and (g) combined global 3D coregistration and 2D regional coregistration. (h) Zoomed views of two sub-areas in stack (e) that highlights the improvements with combined 3D and 2D coregistration. White arrows highlight the improvements by the regional 2D coregistration. All scale bars are 200  μm in length.

Angiogram coregistration was performed by transforming the volumetric intensity images obtained by 2PM to the DLS-OCT space. To account for the non-linearity of image spaces caused by distortions, such as field curvature across those two imaging modalities due to the implementation of different optics, an initial global 3D coregistration was combined with multiple regional 2D coregistrations. The global 3D coregistration was implemented over the whole 3D 2PM and OCT stacks with an affine transformation matrix computed based on >20 manually selected fiducial marker voxels representing the same locations in two stacks. Since 2PM and OCT image distortions change differently along the axial direction, they are difficult to address using global coregistration only. Therefore, coarse coregistration was followed by dividing the stacks along the depth dimension (Z) into a set of 40-μm-thick sub-stacks and conducting 2D coregistration of each pair of sub-stacks. The transformation matrices for 2D local coregistrations were computed from additional fiducial marker voxels manually selected in the sub-stacks after global coregistration. The results of angiogram coregistration for two example sub-stacks selected at different depths are shown in Figs. 4(a)–4(c) and Figs. 4(e)–4(g). Clear improvements are observed from applying combined two-step coregistration compared to using global 3D coregistration only [see Figs. 4(d) and 4(h)]. Herein, we applied a quick and intuitive coregistration method that can better account for the non-linear optical distortions of those two imaging modalities than the methods mainly based on affine coregistration. We also conducted a brief comparison of our method with other coregistration methods, such as the ones based on FreeSurfer,^50^ NiftyReg,^51^^,^^52^ and ANTs^53^^,^^54^ packages. Our method has shown improved coregistration results compared to the MRI_Robust_Register Function from the FreeSurfer package, which is mainly based on affine coregistration and comparable results to the state-of-the-art methods based on NiftyReg and ANTs, which both utilize non-linear deformable coregistration in addition to the affine coregistration (see the Supplementary Material for detailed results of the comparisons). In addition, our method enables a considerable degree of manual, user control over the coregistration process, which may be helpful to deal with sometimes significant deficiencies in acquired datasets, such as large shadows below the vessels or missing data in large sub-ROIs.

### 2.6 Blood Flow Velocity Mapping

**Fig. 5:**
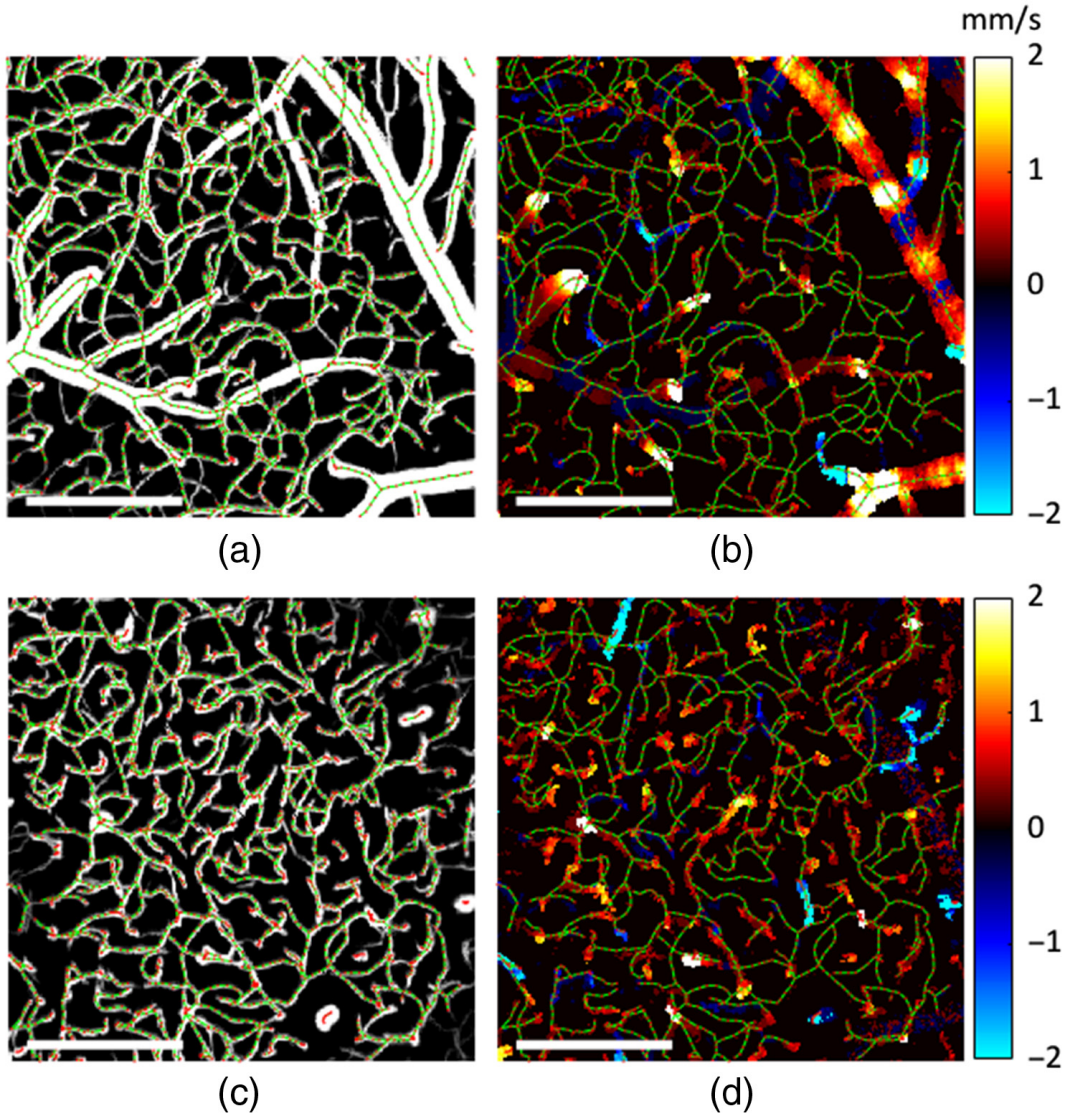
2PM vascular network graphing and coregistration with the DLS-OCT blood flow velocity measurements. The transformed graph of (a) pial and (c) in-depth 2PM stacks superimposed with top MIPs from the corresponding angiogram layers. Co-registered vascular graph and corresponding DLS-OCT axial flow velocity measurements for (b) pial and (d) in-depth stacks. All scale bars are 200  μm in length.

Based on transformation matrices, all nodes from the graph-based network obtained from the 2PM angiogram were co-registered with the DLS-OCT volumes at different depths [Figs. 5(a) and 5(c)]. This enabled mapping of the blood flow velocities obtained by the DLS-OCT to the microvascular segments obtained by the 2PM angiography [Figs. 5(b) and 5(d)]. To assign the blood flow velocities to the microvascular segments represented by the vascular graph, a small cylindrical volume was defined in DLS-OCT space along each graph edge according to the diameter of the corresponding microvascular segment. A mean value of Vz component of the blood flow speed was subsequently extracted from each cylinder. A custom-written software was written in MATLAB (MathWorks, Massachusetts, United States) to enable manual adjustments of cylinder position, orientation, and diameter to correct for the remaining imperfections of coregistration and better match with the DLS-OCT signal from the vessel. In the case of larger vessels (>8  μm diameter) that exhibit parabolic-like flow profile, we extracted the Vz component of the maximum axial velocity in the vessel. The maximum axial velocity in larger vessels typically corresponds to the central region of the vessel. Therefore, the region from which velocities were extracted in such vessels was constrained to an ∼8-μm-diameter cylinder around the center of the vessel. The absolute RBC flow velocities in such vessels were computed using the extracted flow velocities from the cylinder and cosine of the axial tilt angle between the corresponding graph edges and the optical Z axis and defined as mean center-line flow velocities. In the case of capillaries and small arterioles and venules, the extraction region was adjusted to encapsulate most of the vessel cross section since the difference between the RBC velocities from the vessel axis and periphery, if any, could not be resolved in our measurements. Absolute RBC velocities along such vessels were computed similarly using the above method, and the mean center-line flow velocities and mean flow velocities are equivalent for such vessels.

**Fig. 6:**
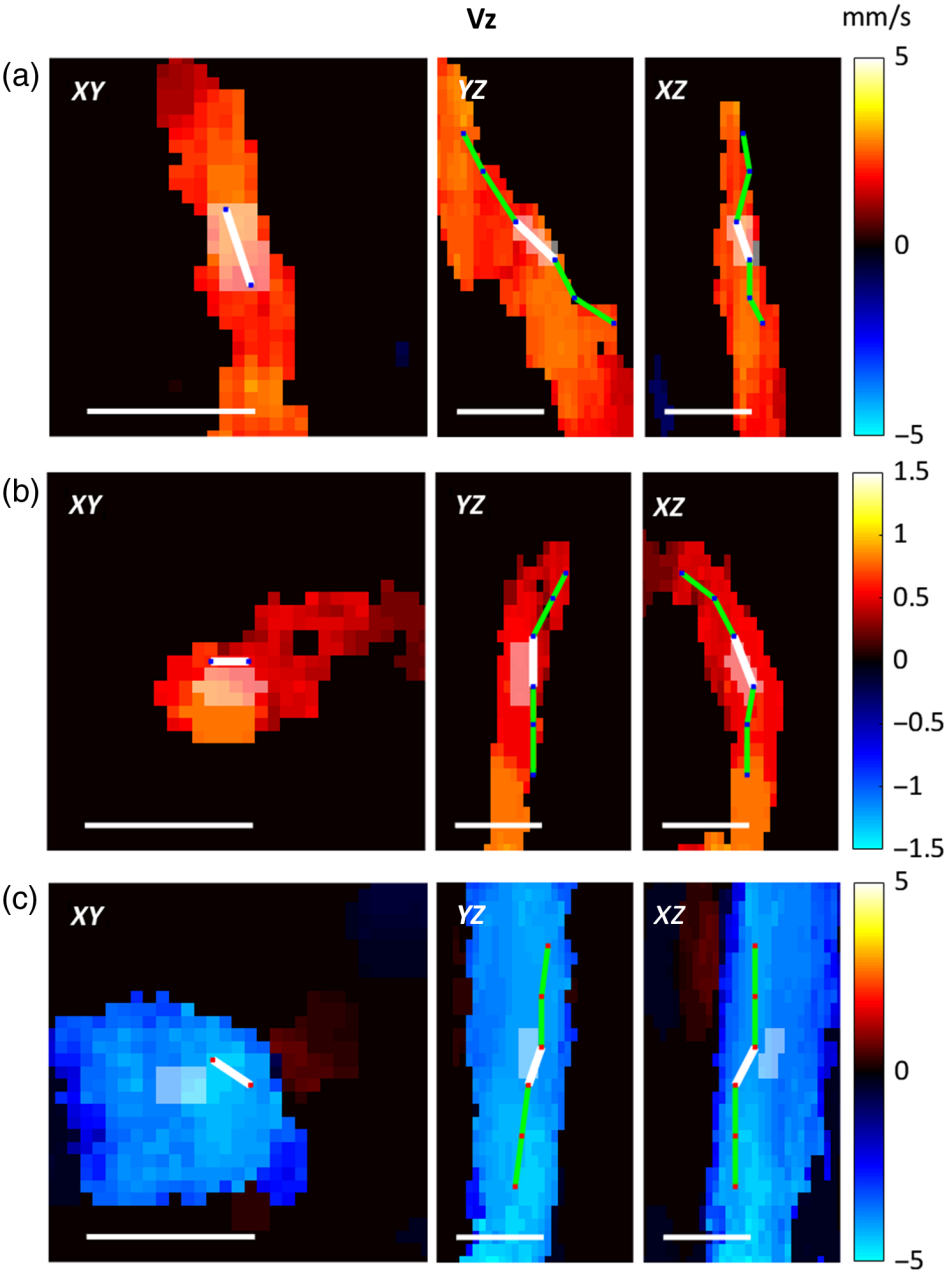
Visualization of axial blood flow velocity (Vz) mapping. The top and side projections of a graph-based vascular segment and its corresponding flow velocity distribution from (a) an artery, (b) a capillary, and (c) a vein. Green lines indicate edges along the segment with a white line segment highlighting the current selected edge. The white shadow areas show projections of the blood flow velocity extraction region after manual corrections. All scale bars are 20  μm in length.

DLS-OCT measurements of the Vz component of the RBC velocity exhibit the largest and the smallest SNR when RBCs are travelling parallel and perpendicular to the optical axis (Z), respectively. Consequently, in the capillary network, Vz velocities were typically too noisy when vessel center-line formed a large axial tilt angle (approximately larger than 50 deg, see the Supplementary Material for details) and led to a pronounced decrease in the goodness of DLS-OCT flow velocity fitting. Therefore, we chose 35 deg as a conservative threshold for the axial tilt angle of capillary edges in data analysis to maintain a high-accuracy standard in DLS-OCT measurements while still enabling the measurements of velocities in a large number of capillary segments. Only the vascular edges with strong DLS-OCT signals were included in the data processing. However, vascular segments typically contain tens of graph edges and mean segment velocities could be obtained even if some edges within these segments needed to be removed from analysis. Examples of mapping the blood flow velocities onto a graph-based network are shown in Fig. 6.

Although most data analysis steps have been performed automatically by the custom-written software, a certain amount of manual corrections and adjustments are still needed in the graphing and coregistration procedures for refined blood flow speed mapping, which is time consuming. In the future, machine learning-based methods may be developed to assist with the manual-correction steps.

## 3 Results of *In Vivo* Measurements and Discussion

**Fig. 7:**
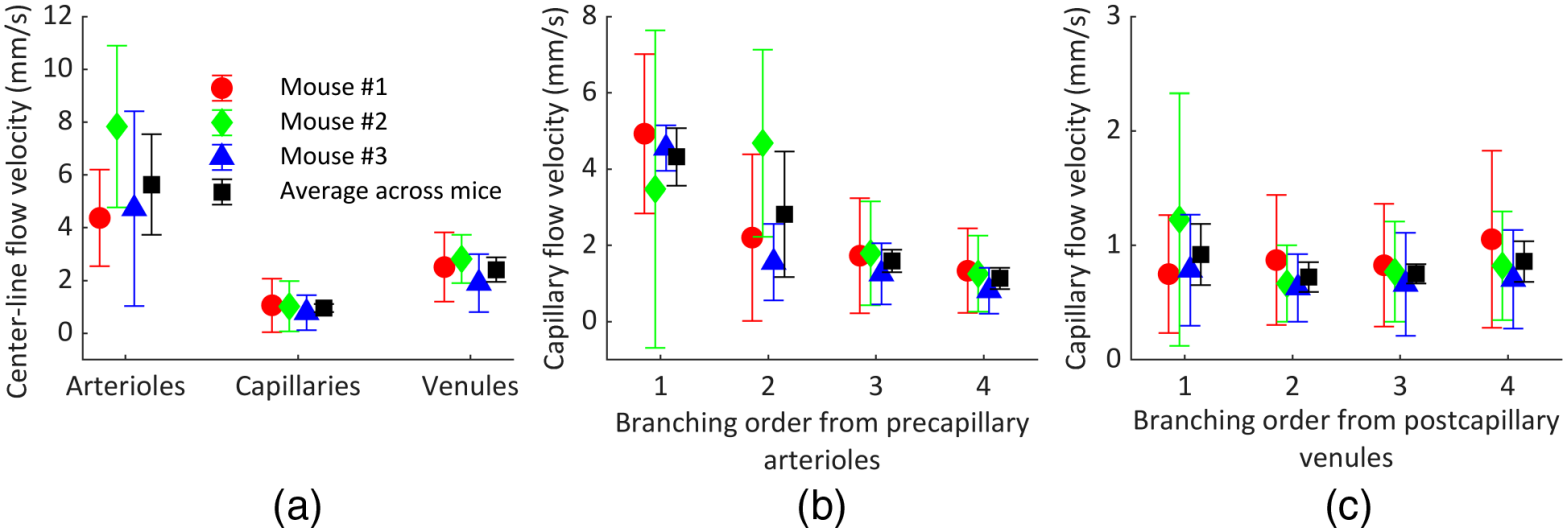
Quantitative analysis of the microvascular velocities obtained from 3 mice. (a) Mean center-line flow velocity distribution in arterioles, capillaries, and venules as labeled with the proposed strategy (454, 316, and 549 vessel segments from mouse #1, #2, and #3, respectively, were used for calculation). Mean flow velocity distributions in capillaries of the first four branching orders from (b) precapillary arterioles and (c) postcapillary venules.

The mean center-line flow velocity distributions in cortical arterioles, capillaries, and venules based on measurements in three mice are shown in Fig. 7(a) and summarized in Table 1. The total numbers of graph edges and segments whose flow velocities were accounted for are 1554 edges/454 segments, 925 edges/316 segments, and 1613 edges/549 segments for mouse #1, #2, and #3, respectively. Herein, using the proposed method, we were able to combine microvascular flow velocities with vascular morphological information on a significantly larger scale than previously reported.^55^ Compared to arterioles, capillaries and venules exhibited 82.1% and 57.1% lower average center-line flow velocities. This is in agreement with previously published measurements^55^ and expected in the mouse cortex as there is larger collective cross-sectional area of venules than arterioles, and even larger collective cross-sectional area of capillaries than venules.

**Table 1:** Mean center-line blood flow velocities in arterioles, capillaries, and venules.

	Mean center-line blood flow velocity (mm/s)		
	Arterioles	Capillaries	Venules
Mouse #1	4.4 ± 1.8	1.1 ± 1.0	2.5 ± 1.3
Mouse #2	7.8 ± 3.1	1.0 ± 1.0	2.8 ± 0.9
Mouse #3	4.7 ± 3.7	0.8 ± 0.7	1.9 ± 1.1
Average across mice	5.6 ± 1.9	1.0 ± 0.2	2.4 ± 0.5

The mean flow velocity distributions in capillaries of the first four branching orders from precapillary arterioles and postcapillary venules are shown in Figs. 7(b) and 7(c) and summarized in Table 2, respectively. Due to truncation of the microvascular networks at the boundaries of the imaged volumes, the assignment of the capillary branching order was reliable only for the first 3 to 4 branching orders from either precapillary arterioles or postcapillary venules. A decreasing trend of capillary flow velocity was observed at the first four branching orders following precapillary arterioles (from 4.3 to 1.1  mm/s), whereas mean flow velocity was similar across the capillaries of the first four branching orders counted from the postcapillary venules (∼0.8  mm/s). A similar trend was previously observed in mice, where average RBC speed in capillaries decreased from ∼3.5 to ∼2.2  mm/s for the first four branching orders following precapillary arterioles while it remained ∼1.0  mm/s for capillaries in the vicinity of postcapillary venules.^55^ This trend of rapid capillary flow speed decrease within the first several branching orders following the feeding penetrating arteriole can be explained by successive vessel bifurcation leading to fast increase of collective cross-sectional area. However, this trend gradually fades away toward the postcapillary venules due to a highly interconnected capillary bed.^56^

**Table 2:** Mean flow velocities in capillaries of the first four branching orders from precapillary arterioles and postcapillary venules.

	Mean blood flow velocity (mm/s)							
	Capillary branching order from precapillary arterioles				Capillary branching order from postcapillary venules			
	1	2	3	4	1	2	3	4
Mouse #1	4.9 ± 2.1	2.2 ± 2.2	1.7 ± 1.5	1.3 ± 1.1	0.7 ± 0.5	0.9 ± 0.6	0.8 ± 0.5	1.1 ± 0.8
Mouse #2	3.5 ± 4.2	4.7 ± 2.5	1.8 ± 1.4	1.3 ± 1.0	1.2 ± 1.1	0.7 ± 0.3	0.8 ± 0.4	0.8 ± 0.5
Mouse #3	4.6 ± 0.6	1.6 ± 1.0	1.3 ± 0.8	0.8 ± 0.6	0.8 ± 0.5	0.6 ± 0.3	0.7 ± 0.4	0.7 ± 0.4
Average across mice	4.3 ± 0.8	2.8 ± 1.6	1.6 ± 0.3	1.1 ± 0.3	0.9 ± 0.3	0.7 ± 0.1	0.8 ± 0.1	0.9 ± 0.2

## 4 Conclusion

We have proposed a method to combine the high-resolution cerebral microvascular angiograms obtained by 2PM and large-scale microvascular blood flow velocity measurements based on DLS-OCT for a comprehensive analysis of cerebral microvascular blood flow network down to capillary level in mice. The developed methodology was applied in proof-of-principle experiments to retrieve the distributions of mean blood flow velocities from several hundred arterioles, capillaries, and venules *in vivo*. We anticipate that this technique will be helpful for quantifying the microvascular blood flow velocity distributions in a broad range of studies involving normal brain functioning, progression of various microvascular diseases, and numerical modeling of the oxygen advection and diffusion in the realistic microvascular networks.^57^

## Code, Data, and Materials Availability

Data and codes used in the work are available online (https://github.com/qipianmgh/MicroscopicFlowMapping) or upon request.
