# Supplementary material for: Cortical microvascular blood flow velocity mapping by combining dynamic light scattering optical coherence tomography and two-photon microscopy
Source: J Biomed Opt. 2023 Jul 21;28(7):076003. doi: 10.1117/1.JBO.28.7.076003 (PMC10362155; doi:10.1117/1.JBO.28.7.076003)
Supplement: Supplementary file 1 [file JBO_028_076003_SD001.pdf]

Cortical microvascular velocity mapping by combining Dynamic Light Scattering Optical Coherence Tomography and Two-photon Microscopy: supplemental document

Comparison of coregistration results between the proposed and other published methods

In this work, we proposed a coregistration module for two-photon microscopy (2PM) and dynamic light scattering optical coherence tomography (DLS-OCT) angiograms as part of the framework to map DLS-OCT flow velocity values to the corresponding cerebral vascular anatomy. We aim at providing a quick and intuitive coregistration method that can better account for the non-linear optical distortions of those two imaging modalities than the method mainly based on affine coregistration like the mri_robust_register function of the FreeSurfer[50] (https://surfer.nmr.mgh.harvard.edu/, version: 7.3.2) package and generate comparable results to state-of-art methods that provide nonlinear deformable coregistration in addition to the affine coregistration such as the ones using the NiftyReg[51,52] (http://cmictig.cs.ucl.ac.uk/wiki/index.php/NiftyReg, version: 1.3.9) and the Advanced Normalization Tools[53,54] (ANTs) (https://github.com/ANTsX/ANTs, version: 2.4.3) packages. Brief comparisons similar to Fig.4 in the main text of the results using the above packages to the proposed methods are shown here in Fig.S1 to S3.

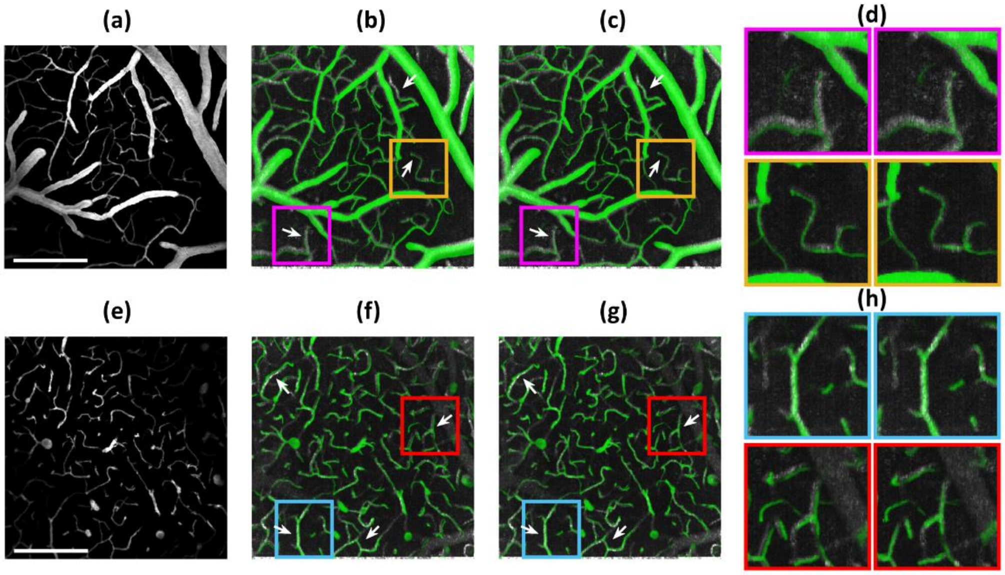

Fig. S1 Comparison of angiogram coregistration results between FreeSurfer affine coregistration and the proposed method. Transformed 2PM pial stack (a) (depth range: 41 μm - 82 μm) in DLS-OCT space and corresponding coregistration results with DLS-OCT angiogram layers (gray) overlaid by the 2PM angiogram (green) using (b) FreeSurfer affine coregistration and (c) combined global 3D coregistration and 2D regional coregistration. (d) Zoomed views of two subareas in stack (a) that compares the two methods. Transformed 2PM in-depth stack (e) (depth range: 157 μm - 197 μm) in DLS-OCT space and corresponding coregistration results with (f) FreeSurfer affine coregistration and (g) combined global 3D coregistration and 2D regional coregistration. (h) Zoomed views of two subareas in stack (e) that compares the two methods. All scale bars are 200 μm in length.

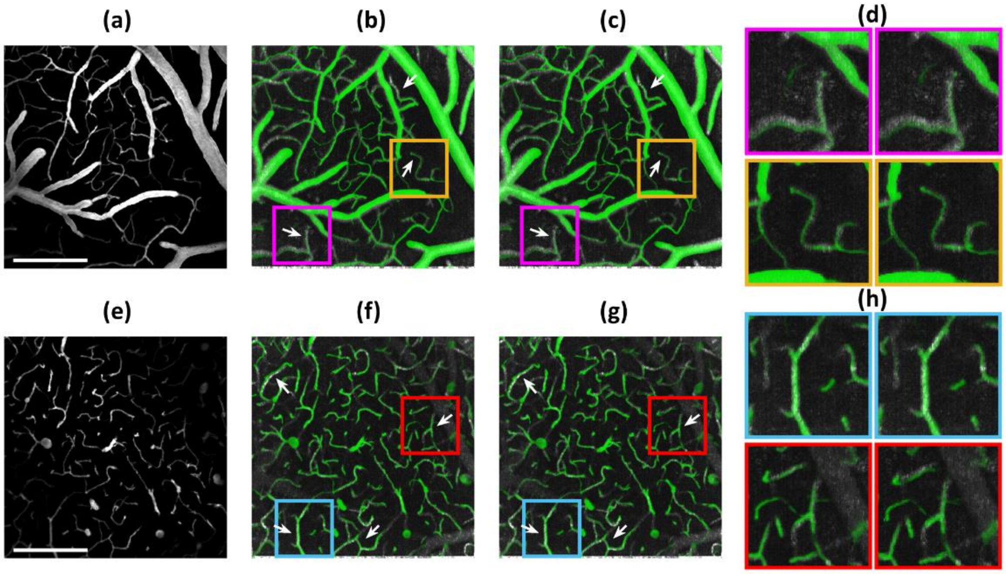

Fig. S2 Comparison of angiogram coregistration results between NiftyReg and the proposed method. Transformed 2PM pial stack (a) (depth range: 41 µm - 82 µm) in DLS-OCT space and corresponding coregistration results with DLS-OCT angiogram layers (gray) overlaid by the 2PM angiogram (green) using (b) NiftyReg and (c) combined global 3D coregistration and 2D regional coregistration. (d) Zoomed views of two subareas in stack (a) that compares the two methods. Transformed 2PM in-depth stack (e) (depth range: 157 µm - 197 µm) in DLS-OCT space and corresponding coregistration results with (f) NiftyReg and (g) combined global 3D coregistration and 2D regional coregistration. (h) Zoomed views of two subareas in stack (e) that compares the two methods. All scale bars are 200 µm in length.

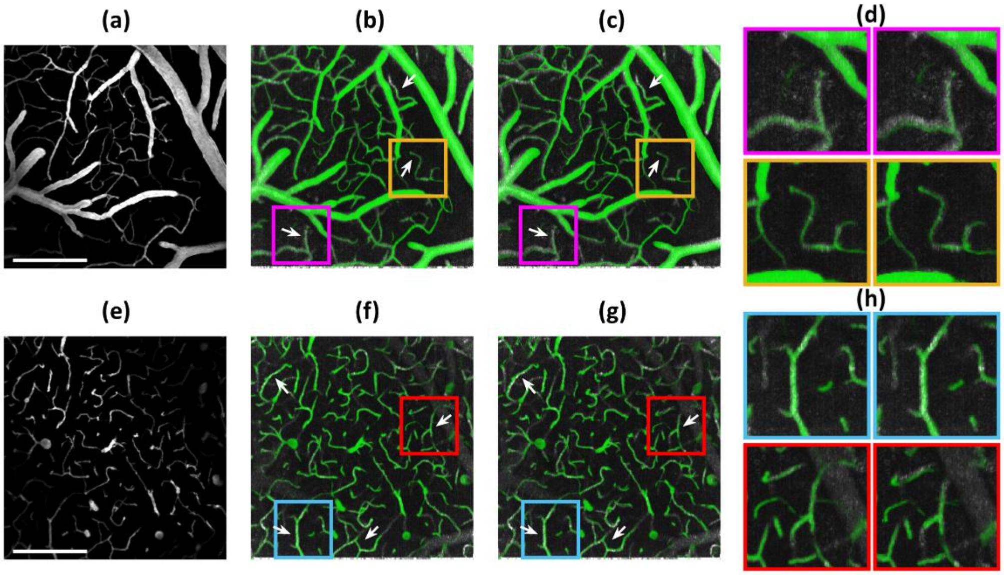

Fig. S3 Comparison of angiogram coregistration results between ANTs and the proposed method. Transformed 2PM pial stack (a) (depth range: 41 µm - 82 µm) in DLS-OCT space and corresponding coregistration results with DLS-OCT angiogram layers (gray) overlaid by the 2PM angiogram (green) using (b) ANTs and (c) combined global 3D coregistration and 2D regional coregistration. (d) Zoomed views of two subareas in stack (a) that compares the two methods. Transformed 2PM in-depth stack (e) (depth range: 157 µm - 197 µm) in DLS-OCT space and corresponding coregistration results with (f) ANTs and (g) combined global 3D coregistration and 2D regional coregistration. (h) Zoomed views of two subareas in stack (e) that compares the two methods. All scale bars are 200 µm in length.

Herein, the affine coregistration of the 2PM and DLS-OCT angiograms using FreeSurfer was repeated twice with each run of mri_robust_register assisted by an initial manual registration step. The reg_aladin and reg_f3d functions of the NiftyReg package were used to perform the affine and deformation transformation respectively for coregistration of the two angiograms and it took ~6 minutes. The antsRegistration function of ANTs package is utilized to perform the two-stage coregistration (affine and diffeomorphic transforms) of the two angiograms and it took ~1 hour (resolution levels = 3; convergence threshold = 1×10^{-6}; physical CPU cores used: 4×Intel Core i7-4790K Processor @ 4.00GHz).

The relationship between the goodness of DLS-OCT blood flow velocity fitting and the axial tilt angle of vessel edges

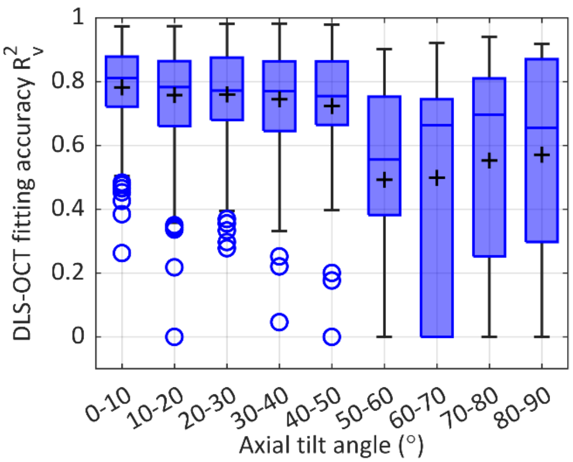

Fig. S4 The relationship between the DLS-OCT blood flow velocity fitting accuracy R_v^2 and the axial tilt angle of vessel edges (Dats from mouse #3 of this study). "+" indicates the average fitting accuracy of each axial tilt angle group.
